# Microbiological Profile and Antimicrobial Resistance Patterns of Endotracheal Aspirates in a Tertiary Care Hospital

**DOI:** 10.7759/cureus.111288

**Published:** 2026-06-22

**Authors:** Malini Jagannatha Rao, Karthik Bhaskaran, Punith Kumar NV, Harika Katepogu

**Affiliations:** 1 Department of Microbiology, Employees' State Insurance Corporation Medical College Post Graduate Institute of Medical Sciences and Research and Model Hospital, Bengaluru, IND; 2 Department of Microbiology, People's Education Society University Institute of Medical Sciences and Research, Bengaluru, IND

**Keywords:** antimicrobial resistance, endotracheal aspirate, healthcare-associated infections, multidrug-resistant organism, non-fermenting gram-negative bacilli

## Abstract

Background

Antimicrobial resistance among Gram-negative bacilli has emerged as a major challenge in hospitalized patients, particularly in respiratory infections associated with prolonged hospitalization and invasive airway devices. Continuous surveillance of respiratory isolates and their antimicrobial susceptibility patterns is essential for guiding empirical therapy and strengthening antimicrobial stewardship practices.

Objective

This study aimed to determine the bacterial profile, antimicrobial susceptibility patterns, and multidrug resistance prevalence among clinical endotracheal aspirate isolates.

Methods

This prospective observational study was conducted in the Department of Microbiology of a tertiary care hospital. Endotracheal aspirate samples received during the study period were processed using standard microbiological techniques. Identification and antimicrobial susceptibility testing of isolates were performed using the VITEK automated system (bioMérieux, Marcy-l'Étoile, France), and results were interpreted according to the Clinical and Laboratory Standards Institute (CLSI) guidelines. Resistance phenotypes including extended-spectrum β-lactamase (ESBL)-associated resistance patterns and carbapenem non-susceptibility among *Enterobacterales *were analyzed.

Results

Of 428 endotracheal aspirate samples processed, 133 (31.1%) yielded significant bacterial growth. Among the culture-positive isolates (n=133), *Acinetobacter baumannii *was the most frequently isolated organism, 48 (36%), followed by *Klebsiella pneumoniae*, 37 (28%), and *Pseudomonas aeruginosa*, 28 (21%). *Acinetobacter baumannii *exhibited extensive multidrug resistance, with amikacin demonstrating the highest susceptibility, 36 (75%), while susceptibility to carbapenems, fluoroquinolones, ceftazidime, and piperacillin-tazobactam remained low. *Pseudomonas aeruginosa *demonstrated moderate susceptibility to amikacin, 19 (67.8%), ciprofloxacin, 13 (46.4%), meropenem, 12 (42.8%), piperacillin-tazobactam, 12 (42.8%), and ceftazidime and imipenem, 11 (39.3%).

Among *Enterobacterales*, 35 (74.5%) demonstrated ESBL-associated resistance patterns, and 37 (78.7%) showed carbapenem non-susceptibility. Colistin retained activity against all *Enterobacterales *isolates tested.

Conclusion

The present study demonstrates a high prevalence of multidrug-resistant Gram-negative bacilli among respiratory isolates obtained from endotracheal aspirate samples, with markedly reduced susceptibility to several routinely used antimicrobial agents. These findings highlight the importance of continuous local antimicrobial surveillance, institution-specific antibiograms, robust infection prevention practices, and strengthened antimicrobial stewardship programs to optimize empirical therapy and limit the emergence of antimicrobial resistance in hospitalized patients.

## Introduction

Healthcare-associated infections (HAIs) remain a major cause of morbidity, mortality, prolonged hospital stay, and increased healthcare expenditure worldwide, particularly among critically ill patients admitted to intensive care units (ICUs) [1,2]. The ICU environment predisposes patients to infections due to the severity of illness, prolonged hospitalization, frequent invasive procedures, immunosuppression, and extensive use of broad-spectrum antimicrobial agents [1]. Among HAIs, lower respiratory tract infections constitute one of the most common and clinically significant infections encountered in critically ill patients [2,3].

Lower respiratory tract infections are among the most common HAIs encountered in ICU patients, particularly among those requiring mechanical ventilation [3,4]. Endotracheal intubation increases the risk of microbial colonization of the lower respiratory tract by bypassing normal host defense mechanisms [5]. Endotracheal aspirate (ETA) culture is a simple, minimally invasive, cost-effective, and commonly used respiratory sampling method for the identification of bacterial pathogens causing lower respiratory tract infections in mechanically ventilated patients [5,6]. Gram-negative bacilli, such as* Klebsiella pneumoniae*, *Pseudomonas aeruginosa*, and *Acinetobacter baumannii*, and other non-fermenting Gram-negative bacteria are commonly isolated from ETAs of ICU patients [7,8].

Increasing antimicrobial resistance among these organisms, including multidrug resistance (MDR), extensively drug-resistant (XDR), and carbapenem resistance, has emerged as a major challenge in ICU settings and a significant global public health concern [7,9]. The widespread and often empirical use of broad-spectrum antibiotics in ICUs has accelerated the emergence of resistant pathogens, thereby limiting therapeutic options and adversely affecting patient outcomes [7-9].

Knowledge of the local microbiological profile and antimicrobial susceptibility patterns of pathogens isolated from ETAs is essential for guiding empirical therapy, formulating antibiotic stewardship policies, and implementing effective infection control practices [3,4]. Continuous surveillance of ICU respiratory pathogens and their resistance trends can contribute significantly to reducing morbidity and mortality associated with hospital-acquired pneumonia (HAP) and ventilator-associated pneumonia (VAP).

Therefore, the present study was undertaken to identify the bacterial pathogens isolated from ETA samples received in the microbiology laboratory and to analyze the antimicrobial susceptibility pattern and prevalence of multidrug-resistant bacterial isolates obtained from ETAs.

## Materials and methods

Study design, setting, and population

This is a hospital-based prospective observational study conducted in the Department of Microbiology of Employees' State Insurance Corporation Medical College Post Graduate Institute of Medical Sciences and Research and Model Hospital, a tertiary care hospital located in Bengaluru, Karnataka, India, to identify bacterial pathogens from ETA samples and determine their antimicrobial susceptibility patterns and prevalence of multidrug-resistant organisms. It was approved by the institute's Institutional Ethics Committee (approval number: 532/L/11/12/Ethics/ESICMC&PGIMSR/Estt.Vol.IV/178-B/2024).

Study duration

The study was conducted over a period of five months from January 2026 to May 2026.

Study sample

The eligibility of study samples (ETA) was determined based on predefined inclusion and exclusion criteria as detailed in Table 1.

**Table 1:** Inclusion and exclusion criteria

Criteria type	Criteria
Inclusion	Endotracheal aspirate samples received in the microbiology laboratory from various inpatient departments, including intensive care units, irrespective of prior antibiotic exposure
Exclusion	Duplicate samples from the same patient
	Improperly collected specimens, defined as samples received in inappropriate or non-sterile containers, specimens with insufficient volume for processing, leaking or damaged containers
	Inadequately labeled specimens: unlabeled or mislabeled specimens

Sample collection and processing

ETA samples were collected aseptically (by the clinician) using a sterile suction catheter from mechanically ventilated patients and transported immediately to the microbiology laboratory for processing. A minimum sample volume of approximately 1-2 mL of ETA was considered adequate for processing. Gram staining was performed for preliminary microscopic examination. Subsequently, the specimens were cultured on blood agar and MacConkey agar plates and incubated aerobically at 37°C for 18-24 hours. Bacterial isolates were identified based on colony morphology, Gram staining characteristics, and standard biochemical tests [10].

Preliminary antimicrobial susceptibility testing was performed using the Kirby-Bauer disc diffusion method on Mueller-Hinton agar, and results were interpreted according to the Clinical and Laboratory Standards Institute (CLSI) guidelines, M100, 35th edition [11]. Isolates identified as non-fermenting Gram-negative bacilli and multidrug-resistant organisms were further processed using the VITEK 2 automated system (bioMérieux, Marcy-l'Étoile, France) for confirmation of identification and antimicrobial susceptibility testing. Quality control for antimicrobial susceptibility testing was performed using standard American Type Culture Collection (ATCC) strains such as* Escherichia coli* ATCC 25922 and* Staphylococcus aureus *ATCC 25923. MDR was defined as resistance to at least one agent in three or more classes of antimicrobial agents.

Data collection and statistical analysis

Patient demographic details, culture findings, bacterial isolates, and antimicrobial susceptibility patterns were recorded and entered into Microsoft Excel (Microsoft Corporation, Redmond, Washington, United States) for analysis. The results were expressed as frequencies, percentages, and proportions wherever applicable.

## Results

A total of 428 ETA samples were received during the study period. Among these, 133 (31.1%) samples yielded significant bacterial growth (mono-microbial), while 295 (68.9%) samples showed no growth or sterile culture findings (Table 2).

**Table 2:** Culture results of endotracheal aspirate (n=428)

Parameter	n (%)
Total endotracheal aspirates received	428 (100)
Culture positive	133 (31.1)
Sterile/no growth	295 (68.9)

Among the 133 culture-positive isolates, the majority of ETA specimens were obtained from patients admitted to ICUs, accounting for 115 (86.4%) isolates. ETAs from medical specialty wards and surgical specialty wards contributed to nine each (6.8%) (Table 3).

**Table 3:** Distribution of culture-positive isolates by location (n=133)

Location	n=133 (%)
Intensive care unit	115 (86.4)
Medicine specialty	9 (6.8)
Surgical specialty	9 (6.8)

Among the 133 culture-positive isolates, *Acinetobacter baumannii *was the predominant organism isolated, accounting for 48 (36%), followed by *Klebsiella pneumoniae *in 37 (28%) isolates and *Pseudomonas aeruginosa *in 28 (21%) isolates. *Escherichia coli *constituted 10 (7.5%) isolates, while other organisms, including *Enterococcus*, *Proteus *spp., and *Streptococcus *spp., also collectively accounted for 10 (7.5%) isolates (Table 4).

**Table 4:** Organism-wise distribution among culture-positive endotracheal aspirates (n=133)

Organism	n (%)
Acinetobacter baumannii	48 (36)
Klebsiella pneumoniae	37 (28)
Pseudomonas aeruginosa	28 (21)
Escherichia coli	10 (7.5)
Others (*Enterococcus*, *Proteus*, *Streptococcus*, etc.)	10 (7.5)
Total	133

Among *Enterobacterales *isolates, *Escherichia coli *(n=10) demonstrated relatively higher susceptibility to aminoglycosides, with 80% susceptibility to both amikacin and gentamicin. However, reduced susceptibility was observed to third-generation cephalosporins and fluoroquinolones. *Klebsiella pneumoniae *showed markedly reduced susceptibility to most commonly used antibiotics, particularly cephalosporins, fluoroquinolones, and beta-lactam/beta-lactamase inhibitor combinations. Colistin demonstrated 100% susceptibility among both *Escherichia coli *and *Klebsiella pneumoniae *isolates (Table 5).

**Table 5:** Antimicrobial susceptibility patterns of Enterobacterales: Escherichia coli (n=10) and Klebsiella pneumoniae (n=37)

Antibiotic	*Escherichia coli *susceptibility, n=10 (%)	*Klebsiella pneumoniae *susceptibility, n=37 (%)
Amikacin	8 (80)	18 (48.6)
Gentamicin	8 (80)	15 (40.5)
Ciprofloxacin	1 (10)	4 (10.8)
Cefotaxime	1 (10)	1 (2.7)
Ceftazidime	1 (10)	1 (2.7)
Amoxicillin-clavulanate	3 (30)	1 (2.7)
Piperacillin-tazobactam	3 (30)	1 (2.7)
Imipenem	5 (50)	8 (21.6)
Meropenem	6 (60)	7 (18.9)
Cotrimoxazole	3 (30)	8 (21.6)
Colistin	10 (100)	37 (100)

Among non-fermenting Gram-negative bacilli, *Pseudomonas aeruginosa *demonstrated moderate susceptibility to amikacin, 19 (67.8%), ciprofloxacin, 13 (46.4%), meropenem, 12 (42.8%), piperacillin-tazobactam, 12 (42.8%), and ceftazidime and imipenem, 11 (39.3%). In contrast, *Acinetobacter baumannii *demonstrated the highest susceptibility to amikacin, 36 (75%), and markedly low susceptibility to most antimicrobial agents tested, including carbapenems and beta-lactam/beta-lactamase inhibitor combinations (Table 6).

**Table 6:** Antimicrobial susceptibility patterns of Pseudomonas aeruginosa (n=28) and Acinetobacter baumannii (n=48)

Antibiotic	*Pseudomonas aeruginosa* susceptibility, n=28 (%)	*Acinetobacter baumannii *susceptibility, n=48 (%)
Amikacin	19 (67.8)	36 (75)
Ceftazidime	11 (39.3)	6 (12.5)
Ciprofloxacin	13 (46.4)	4 (8.3)
Gentamicin	-	17 (35.4)
Imipenem	11 (39.3)	5 (10.4)
Meropenem	12 (42.8)	5 (10.4)
Piperacillin-tazobactam	12 (42.8)	4 (8.3)
Tetracycline	-	14 (29.1)
Cotrimoxazole	-	4 (8.3)

Analysis of resistance patterns among *Enterobacterales *revealed high levels of antimicrobial resistance. Carbapenem non-susceptibility was observed in 37 (78.7%) isolates, while extended-spectrum β-lactamase (ESBL) production was detected in 35 (74.5%) isolates. Amikacin non-susceptibility was identified in 17 (36.2%) isolates (Table 7).

**Table 7:** Resistance patterns among Enterobacterales (includes Escherichia coli and Klebsiella pneumoniae) (n=47)

Resistance pattern	n (%)
Carbapenem non-susceptible	37 (78.7)
Extended-spectrum β-lactamase-producing isolates	35 (74.5)
Amikacin non-susceptible	17 (36.2)

## Discussion

Lower respiratory tract infections associated with endotracheal intubation and mechanical ventilation remain a major cause of morbidity and mortality in hospitalized patients, particularly in intensive care settings. These infections are strongly associated with prolonged hospital stay, increased healthcare costs, and adverse clinical outcomes, especially when caused by multidrug-resistant organisms.

The burden of antimicrobial resistance in such settings has been extensively documented in global surveillance reports, including the World Health Organization (WHO) Global Antimicrobial Resistance and Use Surveillance System (GLASS) data, which highlight the rising prevalence of resistant Gram-negative pathogens in hospital environments [12].

A total of 428 ETA samples were received during the study period. Among these, 133 (31.1%) samples yielded significant bacterial growth (mono-microbial), while 295 (68.9%) samples showed no growth or sterile culture findings (Table 2).

A major proportion of culture-positive isolates (numbering 133) in the present study were obtained from patients admitted to ICUs, accounting for 115 (86.4%) of isolates, while medical and surgical wards contributed only nine (6.8%) each (Table 3).

Although the inclusion criteria of the present study encompassed all ETA samples received by the laboratory regardless of the ward, this marked predominance of ICU-derived isolates reflects the higher burden of healthcare-associated respiratory infections in critically ill patients. This is likely due to factors such as prolonged hospitalization, invasive airway devices, mechanical ventilation, and frequent exposure to broad-spectrum antibiotics.

This high ICU representation explains the increased prevalence of multidrug-resistant organisms observed in this study, as intensive care environments are known to facilitate the selection and transmission of resistant pathogens under strong antimicrobial pressure.

Spectrum of pathogens in ETA specimen

Among the 133 culture-positive ETA isolates, *Acinetobacter baumannii *was the predominant organism isolated, accounting for 48 (36%) isolates, followed by *Klebsiella pneumoniae*, 37 (28%), and *Pseudomonas aeruginosa*, 28 (21%). *Escherichia coli *constituted 10 (7.5%) isolates, while other organisms, including *Enterococcus *spp., *Proteus *spp., and *Streptococcus *spp., collectively accounted for 10 (7.5%) isolates (Table 4). The present study demonstrates a high prevalence of non-fermenting Gram-negative bacilli and *Enterobacterales *among bacterial isolates recovered from ETA samples of patients. These findings reflect the evolving epidemiology of nosocomial pneumonia and are consistent with previously reported ICU-based infection studies [13] and established literature on Gram-negative hospital-acquired pathogens [14].

The predominance of Gram-negative bacilli, particularly non-fermenting Gram-negative bacilli, reflects their strong adaptation to hospital environments and their frequent association with respiratory infections in hospitalized and mechanically ventilated patients. This pattern is consistent with global ICU data, which identifies these organisms as leading Gram-negative pathogens in mechanically ventilated patients, particularly in the setting of prolonged hospitalization and prior exposure to broad-spectrum antibiotics [14,15].

The predominance of *Pseudomonas *spp. and *Acinetobacter baumannii *observed in the present study is consistent with several Indian and international studies evaluating ETA cultures, where these organisms have been identified as major respiratory pathogens, particularly in intensive care settings [13-17]. Their high prevalence is likely related to prolonged hospitalization, invasive airway instrumentation, mechanical ventilation, and extensive exposure to broad-spectrum antimicrobial agents.

The ability of *Pseudomonas aeruginosa *to persist in hospital settings is primarily related to its intrinsic resistance mechanisms, including low outer membrane permeability, multidrug efflux pumps, quorum sensing-regulated biofilm formation, and inducible β-lactamase production. These factors collectively contribute to its persistence in ICU respiratory environments and reduced antimicrobial susceptibility [14]. Similarly, *Acinetobacter baumannii *demonstrates remarkable environmental survival and persistence on hospital surfaces and respiratory devices because of its ability to form biofilms and acquire multiple resistance determinants. Its MDR is mediated by mechanisms including efflux pump overexpression, outer membrane protein alterations, and production of β-lactamases, including carbapenemases, which significantly limit therapeutic options [14,15].

*Enterobacterales*, particularly *Klebsiella pneumoniae *and *Escherichia coli*, also constituted a significant proportion of isolates in the present study. Their presence reflects the continuing burden of multidrug-resistant *Enterobacterales *in respiratory infections among hospitalized patients and is supported by both Indian and global surveillance data [12,14].

Indian studies by Prajapati et al. and Aravind and Jeya similarly documented a predominance of Gram-negative bacilli in ETA cultures, with *Enterobacterales *and non-fermenters constituting the major respiratory pathogens in tertiary care settings [16,17].

Indian ICU-based studies have similarly documented a high prevalence of multidrug-resistant *Enterobacterales*, particularly *Klebsiella pneumoniae*, contributing significantly to respiratory infections with reduced susceptibility to third-generation cephalosporins and increasing reliance on carbapenems [18,19]. A substantial proportion of these organisms are known to produce ESBLs, which confer resistance to third-generation cephalosporins and significantly limit therapeutic options in hospitalized patients. ESBL-producing *Enterobacterales *are widely recognized as important nosocomial pathogens associated with increased morbidity, prolonged hospitalization, and treatment failure, particularly in intensive care settings [20].

Antimicrobial resistance patterns

Non-fermenting Gram-Negative Bacilli

In the present study, a concerning level of antimicrobial resistance was observed among non-fermenters. In particular,* Pseudomonas aeruginosa* demonstrated reduced susceptibility to several commonly used antipseudomonal agents, including piperacillin-tazobactam, 12 (42.8%), ceftazidime, 11 (39.3%), ciprofloxacin, 13 (46.4%), imipenem, 11 (39.3%), and meropenem, 12 (42.8%), corresponding to resistance rates ranging approximately from 52% to 61.5% for these first-line agents. Amikacin showed comparatively higher susceptibility, 19 (67.8%) (Table 6). This trend is consistent with established literature describing increasing MDR among ICU-associated Gram-negative pathogens and the global rise of carbapenem-resistant organisms [14,21].

*Acinetobacter baumannii *demonstrated particularly high levels of MDR in the present study, with markedly reduced susceptibility to commonly used antimicrobial agents, including piperacillin-tazobactam, four (8.3%), ciprofloxacin, four (8.3%), ceftazidime, six (12.5%), imipenem and meropenem, five (10.4%), and cotrimoxazole, four (8.3%). Aminoglycosides showed comparatively lower resistance, with amikacin susceptibility at 36 (75%) and gentamicin susceptibility at 17 (35.4%), while tetracycline resistance was at 14 (29.1%) (Table 6). Overall, amikacin retained the highest activity against non-fermenting Gram-negative bacilli, whereas most routinely used agents demonstrated very limited effectiveness.

This profound resistance profile among ETA non-fermenters is consistent with local literature, as Aravind and Jeya similarly reported extensive resistance to beta-lactams and carbapenems among respiratory isolates, highlighting the diminishing utility of conventional empirical regimens [17]. The ability of *Acinetobacter baumannii *to acquire carbapenemase genes and persist in hospital environments contributes significantly to outbreaks and endemic colonization in healthcare settings [14,15]. Carbapenem-resistant *Acinetobacter baumannii *therefore represents a major therapeutic challenge because of the limited availability of effective treatment options.

Enterobacterales

Among *Enterobacterales *isolates, *Escherichia coli *demonstrated relatively higher susceptibility to aminoglycosides, with eight (80%) isolates susceptible to both amikacin and gentamicin. However, reduced susceptibility was observed to third-generation cephalosporins and fluoroquinolones, with only one (10%) isolate susceptible to cefotaxime and ceftazidime and one (10%) isolate susceptible to ciprofloxacin. *Klebsiella pneumoniae *showed markedly reduced susceptibility to most commonly used antibiotics, including cefotaxime, one (2.7%), ceftazidime, one (2.7%), ciprofloxacin, four (10.8%), and piperacillin-tazobactam, one (2.7%). Colistin demonstrated 100% susceptibility among both *Escherichia coli*,* *10 (100%), and *Klebsiella pneumoniae*, 37 (100%) (Table 5).

*Klebsiella pneumoniae* demonstrated a high level of MDR, with markedly reduced susceptibility to several commonly used antimicrobial agents. Similar findings have been reported by Prajapati et al., who observed substantial resistance among *Klebsiella *isolates recovered from respiratory specimens, particularly to cephalosporins, beta-lactam/beta-lactamase inhibitor combinations, and carbapenems [16]. The severe compromise of carbapenem activity observed in the present study represents a significant therapeutic concern, as these agents have traditionally served as important treatment options for multidrug-resistant Gram-negative infections.

Among *Enterobacterales*, reduced susceptibility to third-generation cephalosporins and carbapenems suggests the presence of ESBL and possibly carbapenemase-producing strains. ESBL-producing organisms are associated with increased mortality, prolonged hospitalization, and higher healthcare costs [21,22].

The increasing resistance in *Klebsiella pneumoniae *is particularly concerning, as carbapenem-resistant *Klebsiella pneumoniae *(CRKP) has been classified by WHO as a critical priority pathogen requiring urgent attention because of its rapid dissemination and limited therapeutic options [12,22].

A notable proportion of *Enterobacterales *isolates in the present study demonstrated important resistance phenotypes based on VITEK-derived susceptibility patterns, with 35 (74.5%) isolates of *Enterobacterales *showing ESBL-associated resistance patterns and 37 (78.7%) demonstrating carbapenem non-susceptibility. In contrast, amikacin non-susceptibility was comparatively lower, 17 (36.2%), indicating retained activity against a proportion of *Enterobacterales *isolates (Table 7). These findings indicate a substantial burden of multidrug-resistant *Enterobacterales *among respiratory isolates obtained from ETAs and suggest reduced effectiveness of third-generation cephalosporins and carbapenems in the study setting. The increasing prevalence of ESBL-associated and carbapenem-non-susceptible *Enterobacterales *is clinically significant because these organisms are associated with limited therapeutic options and increased healthcare burden [12,21,22].

Overall, the antimicrobial resistance patterns observed in the present study are comparable with findings from both Indian and international studies evaluating respiratory isolates from hospitalized patients. The high prevalence of multidrug-resistant Gram-negative bacilli, particularly carbapenem-resistant non-fermenters and *Enterobacterales*, reflects the growing global challenge of antimicrobial resistance in healthcare settings and highlights the importance of continuous local surveillance and antimicrobial stewardship measures.

Role of automated susceptibility testing (VITEK 2 system)

In this study, antimicrobial susceptibility testing was performed using an automated system (VITEK 2). Automated systems provide rapid, standardized, and reproducible minimum inhibitory concentration (MIC)-based results, making them highly useful in clinical microbiology laboratories. However, interpretation must strictly follow the latest CLSI guidelines to ensure accuracy and clinical relevance [11].

While VITEK 2 is generally reliable, confirmatory testing may be required in specific situations, particularly for carbapenem-resistant isolates or when unusual resistance patterns are detected. This is crucial for infection control interventions and antimicrobial stewardship decision-making [11,22].

Clinical implications

The findings of the present study have important implications for antimicrobial stewardship and empirical treatment strategies in hospitalized patients with respiratory infections. The high prevalence of multidrug-resistant organisms necessitates careful selection of empirical antibiotics, particularly in intensive care settings where resistance rates are often elevated.

Empirical therapy should be guided by institutional antibiograms rather than international guidelines alone, as antimicrobial susceptibility patterns vary considerably across hospitals and geographic regions. Early de-escalation of therapy based on culture and susceptibility results remains essential to minimize unnecessary broad-spectrum antimicrobial exposure and reduce selective pressure for antimicrobial resistance [21,22].

The markedly reduced susceptibility observed among both non-fermenting Gram-negative bacilli and *Enterobacterales *highlights the limited effectiveness of many routinely used antimicrobial agents. These findings emphasize the importance of judicious antimicrobial use and, in selected severe infections, the consideration of combination therapy or reserve agents such as polymyxins under appropriate clinical guidance [22].

Infection control and antimicrobial stewardship

The increasing burden of multidrug-resistant organisms underscores the critical importance of infection prevention and control strategies in healthcare settings, particularly in ICUs. Measures such as strict hand hygiene compliance, environmental decontamination, adherence to ventilator care practices, and appropriate device management have been shown to reduce healthcare-associated respiratory infections and transmission of resistant organisms [14].

Antimicrobial stewardship programs play an equally important role in optimizing antibiotic use, minimizing unnecessary broad-spectrum antimicrobial exposure, and slowing the emergence of resistance. Regular microbiological surveillance and periodic institutional antibiogram updates are essential components of effective antimicrobial stewardship strategies and aid in guiding appropriate empirical therapy [12,21].

Limitations of the study

This study has certain limitations. Being a single-center study, the findings may not be generalizable to other healthcare settings. In addition, antimicrobial resistance was determined using phenotypic methods, including automated VITEK-based susceptibility testing, and molecular characterization of resistance mechanisms such as ESBL, AmpC, and carbapenemase genes was not performed; therefore, genetic determinants of resistance could not be confirmed. The analysis was primarily descriptive in nature, focusing on frequencies and percentages of bacterial isolates and their susceptibility patterns. Future multicenter studies incorporating molecular diagnostics and broader epidemiological surveillance would provide more comprehensive insights into antimicrobial resistance patterns among respiratory isolates from ETAs.

## Conclusions

The present study highlights a high prevalence of multidrug-resistant Gram-negative bacilli among respiratory isolates obtained from ETA samples in intensive care settings, with limited susceptibility to several commonly used antimicrobial agents. Non-fermenting Gram-negative bacilli, particularly *Pseudomonas aeruginosa *and *Acinetobacter baumannii*, demonstrated high levels of resistance to multiple first-line antibiotics, while *Enterobacterales *showed significant resistance to third-generation cephalosporins and carbapenems. These findings underscore the urgent need for strengthened antimicrobial stewardship practices, robust infection prevention and control measures, and continuous local antimicrobial surveillance to guide appropriate empirical therapy and help combat the growing threat of antimicrobial resistance in critical care settings. Gram-negative pathogens predominated among VAP isolates, with a substantial burden of antimicrobial resistance. Continuous microbiological surveillance and antimicrobial stewardship are essential for optimizing management strategies in ICU patients.
